# Correction: The relationship between political instability and stock market performance: An analysis of the MSCI index in the case of Pakistan

**DOI:** 10.1371/journal.pone.0315683

**Published:** 2024-12-09

**Authors:** Zhiying Mai, Hassan Mujtaba Nawaz Saleem, Muhammad Kamran

The affiliation for the third author is incorrect. Muhammad Kamran is not affiliated with #3 but with #2: Department of Management Sciences, The Islamia University of Bahawalpur, Punjab, Pakistan.
